# Early-life and contemporaneous nutritional and environmental predictors of antibody response to vaccination in young Gambian adults

**DOI:** 10.1016/j.vaccine.2012.05.009

**Published:** 2012-07-06

**Authors:** Sophie E. Moore, Anna A. Richards, David Goldblatt, Lindsey Ashton, Shousun Chen Szu, Andrew M. Prentice

**Affiliations:** a MRC Keneba, Medical Research Council Laboratories, Fajara, Gambia; b MRC International Nutrition Group, London School of Hygiene & Tropical Medicine, London, UK; c Institute of Child Health, London, UK; d National Institutes of Health, MD, USA

**Keywords:** Vaccine responses, Immune programming, Birth weight, Infant growth, Gambia

## Abstract

Recent research links nutritional exposures early in life with alterations in functional immunity that persist beyond childhood. Here we investigate predictors of antibody response to polysaccharide vaccines in a cohort of Gambian adults with detailed records from birth and early infancy available. 320 adults were given a single dose of a Vi polysaccharide vaccine for *Salmonella typhi* and a 23-valent capsular polysaccharide pneumococcal vaccine. Anti-Vi antibody levels and antibodies against 4 pneumococcal serotypes (1, 5, 14 and 23F) were measured in serum samples collected at baseline and then 14 days following vaccination and compared to data available from birth and early infancy. Post-vaccination antibody titres to serotype 14 of the pneumococcal vaccine were negatively associated with rate of growth from birth to three months of age, infant weight at 12 months of age and season of birth, but no other associations were observed with early-life exposures. The strongest predictor of antibody levels was pre-vaccination antibody titres, with adult height and serum neopterin levels at time of vaccination also implicated. The current study does not support the hypothesis that nutritional exposures early in life consistently compromise antibody response to polysaccharide vaccines administered in young adulthood.

## Highlights

► We investigate predictors of response to vaccination in young Gambian adults. ► We focus on both early-life and contemporary nutritional and environmental exposures. ► Pre-vaccination antibody titres were a consistent predictor of response to vaccination. ► No other consistent associations were observed.

## 1 Introduction

The inter-relationship between nutritional status and immune function continues to be the focus of research and debate [1,2]. It is well documented that acute and chronic deficiency of both macro- and micro-nutrients results in an impairment to a number of components of the immune system [3] and supplementation with individual micronutrients has proven efficacious as therapy for certain infectious morbidities; for instance vitamin A and measles infection [4], and zinc and diarrhoeal disease [5]. More recent research also suggests that supplementation with specific micronutrients may have non-specific deleterious effects on immune function, with iron [6] and vitamin A [7] specifically implicated. Further work to understand the mechanisms of these effects is required.

In addition to the effects of contemporaneous nutritional status on human immune function, recent evidence from our group and others suggests that nutritional status during fetal life and early infancy may be critical for immune development, with effects persisting into adulthood. Using antibody response to vaccination as a functional indicator of immunity, we have previously shown that adults born of a lower birth weight have a reduced antibody response to a polysaccharide vaccine (Typhim Vi) [8]. This deficit persisted following a second ‘booster’ dose of the vaccine [9] but no such association with size at birth was observed with either a protein (rabies) vaccine [8] or a polysaccharide-conjugate (Hib) vaccine [9]. This differential response suggests an early-life programming effect on the generation of antibodies during a B-cell-dependent immune response.

Much of the programming literature has focused on poor maternal nutrition as the most likely candidate for these early-life effects, and uses low birth weight as a proxy indicator for poor nutrition *in utero*. However, low birth weight may also be predictive of a number of post-natal factors that could also be implicated in defining later disease risk. Recent attention has focused on the association between an infant's rate of growth during early-infancy and later disease risk, with faster rates of post-natal ‘catch-up’ growth implicated as a possible causative factor for certain chronic disease outcomes [10]. The current study was therefore designed to investigate in more detail the relationship between nutritional status early in life and response to vaccination in young adults. Here, we investigate antibody response to two polysaccharide vaccines in a cohort of Gambian adults with detailed anthropometric data available from birth and from early infancy.

## 2 Materials and methods

### 2.1 Study population

Since 1949, the UK Medical Research Council (MRC) has been collecting health and demographic data on the populations of three villages (Keneba, Kantong Kunda and Manduar) in the rural West Kiang region of The Gambia. From 1976, and with the establishment of a permanent field station in Keneba, this data collection has incorporated detailed information on maternal and infant health, including birth anthropometry and infant growth. In the current study, our recruitment pool consisted of all adults, born in the three study villages since 1976 and who were aged 18 years or older on 1st January 2006. Subjects were excluded if they could not be traced or were not accessible for follow up, if they were already enrolled in another MRC study or if they were known to be pregnant at the time of recruitment. Ethical approval for the study was given by the Ethics Committee at the London School of Hygiene and Tropical Medicine and by the joint Gambian Government/MRC The Gambia Ethics Committee. Informed written consent was obtained from each individual participant.

The study took place between February and May 2006. Subjects were seen on two occasions, 14 days apart. At visit 1 (Day 0) weight, height, waist and hip circumferences were measured using standard equipment. A single sample of fasted venous blood was collected for measurement of plasma leptin and serum neopterin: leptin was measured as a proxy marker of adiposity and neopterin as a marker of immune activation. This blood sample was additionally used for the assessment of pre-vaccination serum antibody titres and for the preparation of a thick film for detection of malaria parasites by microscopy. Following blood collection, each subject was given a single dose of a Vi polysaccharide vaccine for *Salmonella typhi* (Sanofi-Pasteur, Lyon, France) and a single dose of a 23-valent capsular polysaccharide vaccine (Pneumo, Sanofi-Pasteur, Lyon, France). Fourteen days later (Visit 2), a further venous blood sample was collected for post-vaccination serum antibody titres.

### 2.2 Laboratory methods

Plasma leptin and serum neopterin were measured at MRC Human Nutrition Research, Cambridge UK. Leptin was measured by ELISA (R&D Systems, Abingdon, UK) and neopterin by a competitive enzyme immunoassay principle (BRAHMS Atiengesellschaft, Berlin, Germany). Both analytes were measured in duplicate and following manufacturers’ guidelines.

Anti-Vi immunoglobulin G (IgG) analysis was conducted at the Laboratory of Developmental and Molecular Immunity, National Institutes of Child Health and Human Development, Bethesda, USA. Briefly, microtitre plates were coated with Vi (0.2 μg/well) purified from *Citrobactor freundii* and goat anti-human IgG (Jackson Immuno Research Laboratories Inc., West Grove, PA) conjugated to alkaline phosphatase were used for ELISA. The anti-Vi IgG standard was a plasma sample from an adult vaccinated with Vi polysaccharide typhoid vaccine (provided by Wendy Keitel, Baylor University, Houston, TX). The Vi antibody content of this serum was also assayed by a radioimmunoassay (RIA) by Pasteur Merieux Connaught. The antibody levels were expressed in ELISA units (EU) and the reference sera were assigned a value of 75 EU. All samples were run in duplicate. Antibody levels were calculated using Program ELISA, version 12 (Center for Disease Control and Prevention, Atlanta, GA). The lowest detectable level of the assay for anti-Vi IgG was 0.1 EU. Prior to analysis, all data were log transformed, and results are presented as geometric means. For anti-Vi antibody levels, data are expressed as ELISA units (EU).

Pneumococcal capsular polysaccharide specific IgG levels were measured at the WHO Pneumococcal Serology Reference Lab at the UCL Institute of Child Health, London, UK. Standard enzyme linked immunosorbent assay methods [11] were used to quantify anticapsular IgG antibodies to four specific pneumococcal serotypes (1, 5, 14 and 23F). These serotypes were selected on the basis of frequency of carriage within this population setting, 14 and 23F being amongst the most common [12], and their importance in causing invasive disease (1 and 5 account for >40% in a recent series of pneumococci causing bacteraemia [13]).

### 2.3 Statistical analyses

Comparisons amongst group means were made using two-sample *t*-tests. Vaccine data are presented as geometric means and 95% confidence intervals (CIs). Sex specific *z*-scores were calculated using UK reference data [14]. Associations between contemporary measures and antibody response to vaccination were compared by linear (for continuous variables) or logistic (for binary variables) regression analysis. Response to vaccination was assessed in relation to six early-life exposures (separate models); birth weight, low birth weight (<2.5 kg) *vs*. normal birth weight (as a binary variable), small for gestational age *vs*. appropriate for gestational age (as a binary variable), rate of infant growth from birth to three months of age, infant weight at 12 months of age and season of birth (harvest/wet season January–June; hungry/dry season July–December). Rate of change in weight from birth to three months was calculated as the difference between sex-specific birth weight standard deviation score and sex-specific weight at three months standard deviation score. We also looked at weight for age standard deviation differences between three and six months of age and six and 12 months of age. Associations between these early-life exposures and antibody responses were tested by multiple linear regression analysis. Probability values <0.05 were considered to be statistically significant for all tests. All statistical analyses were performed using DataDesk, version 6 for Windows, Data Description Inc., Ithaca, NY.

## 3 Results

### 3.1 Subject characteristics

A total of 858 individuals met the criteria for recruitment into the current study. Of these, 78 were known to have died prior to follow up, leaving a cohort of 781 to be traced. Of this number, 145 were excluded on the basis they were currently participating in another ongoing study and three because they were confirmed to be pregnant by an MRC midwife prior to the start of the study. Of the remaining 633 individuals who were eligible to participate, 241 were not available [dead (4), self-confirmed as pregnant (45), overseas (24), outside designated study area (58), not traceable (50), traceable but unavailable for study (60)] and 72 did not consent to participate. A total of 320 subjects (41% of 781 followed up) consented and participated in the current study. Compared to non-participants, participants were younger (22.2 y *vs*. 23.0 y; *p* < 0.0001) and there were significantly more males than females (51.9% *vs*. 45.3%). No differences were observed between the participants and non-participants in available early-life information (data not presented). Table 1 details the early-life characteristics of the subjects recruited. A total of 41 (12.8%) of subjects were born of a low birth weight (<2.5 kg), and a higher proportion of these were female. Of these, 13 were born pre-term (<37 weeks gestation), although 9 had a missing gestational age. A total of 267 (83%) of the cohort had gestational age assessments available. Using the William's reference data [15], 51 (19%) of these infants would be considered small for gestational age (SGA). Male subjects were significantly heavier at three months and at 12 months of age, but the rate of early growth, expressed as the sex-specific change in *z*-score between birth and three months of age, three to six months, or six to twelve months did not differ between males and females.

Characteristics of the study participants at follow up are detailed in Table 2. Male participants were significantly taller and heavier than females, but had a lower mean Body Mass Index (BMI) and plasma leptin level. Significantly more of the males lived in urban areas of The Gambia compared to females, and the distribution of month of study differed between the males and females recruited. No differences were observed in age, waist:hip ratio, or serum neopterin levels between the male and female subjects.

### 3.2 Antibody response to vaccination

Pre- and post-vaccination geometric mean (95% CI) data for both the pneumococcal and Vi vaccine are detailed in Table 3. A total of 112 subjects (37.2%) did not achieve antibody titres >3.52 EU following Vi vaccination, the estimated level for 90% protection. Using a post-vaccination anti-pneumococcal IgG titre of >0.35 μg/mL, the level considered indicative of putative protection, all subjects achieved an adequate response to all serotypes.

Simple univariate regression analysis was used to test for unadjusted associations between antibody response to vaccination and the contemporary variables measured at the time of vaccination; sex, age, location (rural *vs*. urban), weight, height, BMI, plasma leptin, month of study (February, March, April, May), malaria parasitaemia (+ve *vs*. −ve), and serum neopterin levels (Table 4). Pre-vaccination antibody titres were also included as a potential confounder in all of the models. Variables showing significant associations with antibody response to vaccination were then fitted into a multivariate model; those variables that remained significant are as detailed in Table 4. Only those variables that remained significant predictors of antibody response were then added to the models looking at early-life influences on response to vaccination.

We did not predict, *a priori*, that pre-vaccination antibody levels would have such a strong influence on post-vaccination antibody responses. However, and as pre-vaccination levels could themselves be predicted by early life exposures (through immune responses to infection), we repeated the analysis (a) looking at predictors of pre-vaccination levels *per se*, and (b) removing pre-vaccination levels from the final model of predictors of post-vaccination levels. Following adjustment for contemporary factors shown to be associated with pre-vaccination levels, the only significant association observed was between infant weight at 12 months of age and pre-vaccination levels to pneumococcal serotypes 5 and 23 (*p* = 0.028 and 0.016 respectively; analyses not presented). The results of the regression analysis excluding pre-vaccination levels are included in Table 5.

Associations between early-life exposures and antibody responses to vaccination were tested by multiple linear regression analysis, adjusting for the contemporary variables identified as predictive of antibody responses. Table 5 highlights the unadjusted and adjusted results of multiple linear regression analysis using birth weight, low birth weight (<2.5 kg) *vs*. normal birth weight, rate of infant growth from birth to three months of age, infant weight at 12 months of age, and season of birth (hungry *vs*. harvest) as dependent variables (separate models employed for each variable). No significant associations were observed between the early-life data and antibody response to vaccination with either a Vi polysaccharide vaccine or with serotypes 1, 5 and 23f of the pneumococcal polysaccharide vaccine. For serotype 14, no associations were observed with birth weight or low birth weight, but a trend towards significance was observed for infant growth from birth to three months of age (negative trend), infant weight at 12 months of age (negative trend) and season of birth (higher in hungry season births). The analyses were also performed using change in weight-for-age standard deviation scores between three and six, and six and twelve months of age. No significant associations were observed, with the exception of a marginally significant relationship between rate of growth between six and twelve months of age and antibody response to serotype 14, when adjusted for pre-vaccination antibody levels (*β* = −0.116, *p* = 0.043; other data not presented).

## 4 Discussion

Recent research has highlighted a possible association between nutritional status in early-life and development of the human immune system, with long-term programming effects on immune function inferred [16]. Studies in Gambian [17] and Bangladeshi [18] infants have shown correlations between pre- and post-natal nutritional and environmental exposures and development of the thymus during early infancy. In The Gambia, these alterations in thymic size were reflected by changes in both lymphocyte subpopulation counts [19] and in levels of signal-joint T-cell receptor rearrangement circles (sjTREC), an indirect marker of thymic output, suggesting an effect on thymic function [20]. Of importance, this early-life effect appears to persist beyond infancy. Results from studies in adolescents from the Philippines [21] and in adults from Pakistan [8,9] indicate a positive association between birth weight and antibody response to a Vi polysaccharide vaccine for *S. typhi*. In the study in Pakistan, no association however was observed in antibody response to either a rabies (protein) vaccine [8] or polysaccharide conjugate (conjugated *H. influenzae* type b (Hib) vaccine) vaccine [9]. These contrasting effects suggest that antibody generation to polysaccharide antigens, which have greater B-cell involvement, may be compromised by fetal growth retardation.

The current study was specifically designed to explore the relationship between markers of both pre-and post-natal nutritional status and antibody response to polysaccharide antigen vaccines in adults born in rural Gambia. In this cohort of 320 young Gambian adults, no associations were observed between birth weight, low birth weight (<2.5 kg), small for gestational age, rate of growth from birth to three months of age, infant weight at 12 months of age, or season of birth with antibody response to the Vi polysaccharide vaccine or serotypes 1, 5 and 23f of the pneumococcal vaccine. Antibody responses to serotype 14 of the vaccine however were higher amongst infants who were smaller at 12 months of age and showed slower growth between 3 and 12 months of age. In addition, infants born during July to December (the ‘hungry’ season) had higher antibody titres to serotype 14. The data from this study offer only limited support an early-life programming effect of early nutrition on antibody response to vaccination in adulthood within this environment.

The observed associations between early life exposures and response to serotype 14 of the pneumococcal vaccine only are rather difficult to explain. Globally, serotype 14 is the most important serotype causing disease worldwide, although carriage rates vary between populations [12,22,23]. Of the 4 serotypes assessed in the current study (1, 5, 14 and 23f), exposure to 23F and 14 are most likely similar and so early exposures during infancy are unlikely to explain the difference. Technically, type 14 is the ‘purest’ serotype to assay, with little cross-reaction with other serotypes when measured in ELISA (D Goldblatt, personnel communication), but it is unlikely that this alone explains the observed differences. Selection of serotypes was primarily based on carriage rates amongst infants in The Gambia. However, and since it is known that pneumococcal carriage is not equally distributed between adults and children in this population, and is also variable by age (for infants) and season [24], knowledge of precise carriage rates (through nasopharyngeal swabs) at the time of vaccination may have been informative. Inclusion of additional serotypes, such as those known to elicit a ‘weak’ response may help explain this observation. Indeed, previous research has identified serotype 6B as being sensitive to modulation by infant feeding status[25], following vaccination with a conjugated vaccine. Such serotypes may, therefore, be more sensitive to nutritional exposures early in life.

In interpreting the results presented here, consideration should be given to the limitations of the current study. Much of the programming literature in based on the follow up of cohorts of adults for whom records from early-life are available. In The Gambia, the UK Medical Research Council (MRC) has been maintaining demographic and health-related records for three rural villages since 1949 [26]. From 1976, these records have included detailed information on maternal and infant health, allowing the study of early-life predictors of current health within this population. However, as with many studies within this field [27], the current study suffered with considerable loss to follow up. A total of 78 (9%) of the 858 subjects born during the study period were known to have died prior to the start of fieldwork. In addition, we were only able to recruit 41% of the remaining 781 subjects available for follow up. Whilst no differences were observed between the early-life data for those subjects recruited and those available but not included, these subjects only represent the surviving cohort. A major limitation therefore is that the subjects recruited do not provide a true representation of the original cohort; indeed, birth weights amongst subjects who were known to have died prior to follow up were significantly lower than those listed as available for follow up (2.58 kg *vs*. 2.97 kg; ≤0.0001), perhaps indicating that the more vulnerable subjects had already been lost from the cohort. A further limitation of this study design is the lack of any direct measure of early-life nutritional exposures in these subjects, including the assessment of breast feeding practices. Whilst it might be assumed, based on the literature from this population [28,29], that all subjects would have been initially exclusively breast fed, followed by a period of extensive breast feeding, given the literature on the association of early breast feeding practices and later antibody response to vaccination *e.g.* [30], the lack of any detailed information must be viewed as a limitation. Indeed, a strong criticism of much of the programming field is the lack of direct data assessing the impact of nutritional exposures on health outcomes and the reliance on observational data. Future work could usefully focus on cohorts for whom direct measures of early-life nutritional exposures are available, such as the follow-up of randomized control trials of pre- or post-natal nutritional supplementation, and also incorporate more detailed measures of cellular immunity, to help interpret vaccine response data.

To understand the differential results between this study in The Gambia and our previous observations from Pakistan, differences in study design and cohort characteristics need consideration. Firstly, the Gambian adults were significantly younger than the adults in Lahore (mean age 22.3 y *vs*. 29.4 y; *p* ≤ 0.0001) and so it is possible that their relative immaturity contributed to these findings. This, however, seems unlikely since a further study in adolescents from the Philippines (mean age 14.6 y) also observed a positive association between birth weight and antibody response to the same Vi vaccine [21]. In the current study, the geometric mean (GM) post-vaccination anti-Vi antibody titre was 7.1 EU whilst in Pakistan the GM was 5.9 EU (unadjusted difference between means *p* = 0.1383): in both countries, post-vaccination levels were measured 14 days following vaccination. Although this difference in GMs is not statistically significant, it is possible that it may contribute to the lack of an association in the current study, perhaps by suggesting these young Gambian adult were able to mount an overall improved response to vaccination, diminishing the potential impact of the early-life environment. The most consistent predictor of antibody response to vaccination in the current study was pre-vaccination antibody levels. However, pre-vaccination levels were very similar between the two studies (Gambia GM = 0.56 EU, Pakistan GM = 0.53 EU; *p* = 0.8327) and so unlikely to explain the lack of association with birth weight observed in the current study. Relative differences in relation to the pneumococcal vaccine cannot be compared since this vaccine was not used in the study in Pakistan.

In the current study we observed an interesting effect of a number of contemporaneous measures and antibody response to both vaccines. When combined in multiple regression analyses, the measures shown to have the most significant effects were serum neopterin and plasma leptin levels, and pre-vaccination antibody titres. Neopterin is a macrophage-derived protein commonly used as a marker of immune activation, and elevated levels of peripheral blood neopterin indicate an unregulated cellular immune response. In the current study, serum levels of neopterin independently and positively predicted antibody response to serotypes 1 and 5 of the pneumococcal vaccine, but not to serotypes 14 and 23F or the response to the Vi vaccine. Although it is difficult to explain why individuals with elevated immune activation responded more effectively to these two serotypes only, we speculate that an enhanced vaccine response in subjects could be the result of a co-stimulatory effect of an already elevated state of immune activation. Whether such an effect has any longer term implication on antibody titres, remains to be determined.

Leptin, a primarily adipocyte-derived hormone, was positively correlated with serotype 14 of the pneumococcal vaccine but not with the response to any other serotypes or the Vi vaccine. Leptin levels correlate with body fat mass and leptin has more recently been implicated as a central mediator connecting nutrition to immunity [2]. Data from animal models have suggested that leptin may mediate the effects of malnutrition on T cell function [31,32], although little data currently exists to suggest that these effects translate into compromised specific immune responses in malnourished humans (*e.g.* [33]). Further work may be warranted to help understand the specific relationship between plasma leptin levels and antibody response to serotype 14 of the pneumococcal vaccine.

With the exception of antibody response to serotype 23F of the pneumococcal vaccine, a highly significant effect of pre-vaccination antibody levels on post-vaccination titres was observed for both vaccines. Pre-vaccination antibody titres are a consequence of previous exposure to the vaccine antigens; for pneumococcal serotypes this is mainly *via* exposure to the same or similar serotypes encountered during nasopharyngeal carriage. A longitudinal study of households in the UK showed strong immune response to the carriage serotype, supporting the assumption that natural immunity to *Streptococcus pneumoniae* is induced by exposure to *S. pneumoniae* [34].

In conclusion, we have shown that antibody response to 2 polysaccharide vaccines is not influenced by the birth weight, size in infancy or season of birth in rural Gambia, suggesting that young Gambian adults are able to mount an adequate response to both vaccines, irrespective of their early life environment. In addition to the predictive capacity of pre-vaccination antibody levels, these data suggest a role of immune activation and plasma leptin in antibody response to vaccination, but these observations were not consistent between vaccines.

## Figures and Tables

**Table 1:** Early-life variables.

	Males (*n* = 166)	Females (*n* = 154)
Birth weight (kg)	3.10 (0.42)	2.87 (0.41)^a^
Low birth weight (%)	8.43	17.5^b^
Gestational age (wks)	38.9 (1.43)	38.6 (1.56)
Small for gestational age (%)	16.9	21.4
Hungry season births (%)	51.8	53.9
Wt at 3 months (kg)	5.92 (0.84)	5.35 (0.79)^a^
Change in *z*-score, birth to 3 months	0.43 (0.99)	0.36 (0.99)
Change in *z*-score, 3–6 months	−0.56 (0.72)	−0.55 (0.67)
Change in *z*-score, 6–12 months	−0.84 (0.80)	−0.72 (0.88)
Wt at 12 months (kg)	8.28 (1.04)	7.65 (1.03)^a^

All data are means (±SD) or percentages of total. Hungry season = July–December inclusive. Gestational age (and small for gestational age) data only available for *n* = 267 (83%) of total cohort.

a Significantly different from males ≤0.0001.

b Significantly different from males ≤0.05.

**Table 2:** Subject characteristics.

	Males (*n* = 166)	Females (*n* = 154)
Age (y)	22.1 (2.77)	22.6 (3.02)
Weight (kg)	60.8 (8.67)	55.7 (8.69)^a^
Height (cm)	173.3 (722)	160.3 (6.77)^a^
BMI (kg/m^2^)	20.2 (2.19)	21.7 (3.01)^a^
Waist:hip ratio	0.77 (0.04)	0.77 (0.05)
Plasma leptin (ng/mL)^b^	0.73 (0.64–0.84)	9.68 (8.51–11.05)^a^
Rural living (%)	15.1%	31.8%^b^
Month of study F/M/A/M (%)	3.0/21.7/24.7/50.6	18.8/19.5/33.8/27.9^a^
Malaria parasite positive	2/166	0/154
Neopterin (nmol/L)	7.37 (7.10–7.64)	7.51 (7.25–7.79)

All data are means (±SD) or percentages of total. Leptin and neopterin data are reported as geometric means (95% CI). Month of Study data are report as percent of total per month.

a Significantly different from males ≤0.0001.

b Significantly different from males ≤0.001.

**Table 3:** Pre-and post-vaccination antibody concentrations.

	IgG anti Vi antibody concentrations (EU)		IgG anti-pneumococcal antibody concentrations (μg/mL)							
	Pre	Post	Type 1		Type 5		Type 14		Type 23F	
			Pre	Post	Pre	Post	Pre	Post	Pre	Post
*n*	316	301	315	302	316	302	316	302	316	302
GM	0.56	7.19	1.28	10.17	1.92	6.45	8.49	45.5	1.62	7.01
95% CI	0.49–0.63	6.11–8.46	1.18–1.40	9.05–11.4	1.76–2.09	5.83–7.13	7.46–9.67	40.9–50.5	1.45–1.81	6.23–7.88
Non responder		37.2%		0%		0%		0%		0%

GM – geometric mean; EU – Elisa Units. Non-responders: response to Vi vaccination, post-vaccination titres < 3.52 EU considered as a non-responder. For pneumococcal vaccination, post-vaccination titres < 0.35 μg/mL considered as a non-response.

**Table 4:** Associations between contemporary variables and post vaccination antibody concentrations.

Variable	Vi		Type 1		Type 5		Type 14		Type 23F	
	[1]	[2]	[1]	[2]	[1]	[2]	[1]	[2]	[1]	[2]
Sex			*r* = 0.125 *p* = 0.030				*r* = 0.170 *p* = 0.003		*r* = 0.166 *p* = 0.0037	
Age					*r* = 0.133 *p* = 0.021	*β* = −0.041 *p* = 0.006				
Location										
Weight			*r* = −0.160 *p* = 0.005							
Height							*r* = −0.188 *p* ≤ 0.001	*β* = −0.002 *p* = 0.038	*r* = −0.141 *p* = 0.012	
BMI	*r* = 0.171 *p* = 0.0005									
Leptin	*r* = 0.164 *p* = 0.0089		*r* = 0.112 *p* = 0.052							
Month of study										
Malaria										
Neopterin			*r* = 0.153 *p* = 0.0083	*β* = 0.500 *p* = 0.025	*r* = 0.125 *p* = 0.032	*β* = 0.476 *p* = 0.017				
Pre-vaccination antibody levels	*r* = 0.664 *p* ≤ 0.0001	*β* = 0.696 *p* ≤ 0.0001	*r* = 0.566 *p* ≤ 0.0001	*β* = 0.696 *p* ≤ 0.0001	*r* = 0.495 *p* ≤ 0.0001	*β* = 0.544 *p* ≤ 0.0001	*r* = 0.405 *p* ≤ 0.0001	*β* = 0.302 *p* ≤ 0.0001	*r* = 0.559 *p* ≤ 0.0001	*β* = 0.568 *p* ≤ 0.0001

Data in columns labelled [1] represent univariate comparisons between variable and antibody response to vaccination. Data in columns labelled [2] represents output from multivariate analysis where significant variables from [1] were fitted within the same model. Blank cells represent non-significant associations.

**Table 5:** Association between early-life variables and post-vaccination antibody concentrations.

	Vi		Type 1		Type 5		Type 14		Type 23F	
	*β*	*p*-Value	*β*	*p*-Value	*β*	*p*-Value	*β*	*p*-Value	*β*	*p*-Value
Birth weight	−*0.246*	*0.210*	−*0.019*	*0.166*	−*0.056*	*0.645*	*0.015*	*0.905*	−*0.120*	*0.393*
	−0.222	0.124	−0.131	0.262	−0.008	0.938	0.165	0.185	−0.011	0.927
	−0.246	0.210	−0.156	0.266	0.021	0.413	0.015	0.905	−0.120	0.393
Low birth weight	−*0.200*	*0.415*	−*0.156*	*0.375*	−*0.159*	*0.292*	−*0.180*	*0.252*	*0.025*	*0.889*
	−0.008	0.966	−0.051	0.730	−0.139	0.292	−0.272	0.065	−0.090	0.543
	−0.200	0.415	−0.183	0.297	−0.191	0.223	−0.180	0.252	0.025	0.889
Small for gestational age	*0.056*	*0.814*	−*0.032*	*0.849*	−*0.203*	*0.182*	−*0.164*	*0.280*	*0.242*	*0.146*
	0.098	0.568	−0.113	0.416	−0.217	0.100	−0.179	0.187	−0.080	0.584
	0.056	0.814	−0.034	0.837	−0.167	0.274	−0.234	0.117	0.242	0.146
Early catch-up growth	−*0.011*	*0.892*	−*0.025*	*0.674*	−*0.059*	*0.272*	−*0.140*	*0.009*	−*0.006*	*0.920*
	0.027	0.662	0.024	0.630	−0.042	0.391	−0.121	0.015	0.026	0.645
	−0.011	0.892	0.006	0.926	−0.035	0.521	−0.118	0.026	−0.006	0.920
Infant weight at 12 months	−*0.045*	*0.567*	−*0.086*	*0.132*	−*0.071*	*0.151*	−*0.134*	*0.008*	−*0.073*	*0.204*
	−0.026	0.655	−0.019	0.697	−0.025	0.566	−0.102	0.054	0.601	0.876
	−0.045	0.567	−0.058	0.316	−0.090	0.078	−0.134	0.008	−0.073	0.204
Season of birth	*0.075*	*0.652*	*0.147*	*0.218*	*0.048*	*0.642*	*0.234*	*0.028*	−*0.058*	*0.630*
	−0.031	0.802	0.092	0.350	−0.003	0.975	0.223	0.021	−0.076	0.444
	0.075	0.652	0.135	0.254	0.031	0.771	0.234	0.028	−0.058	0.630

Data in italics in first row represents unadjusted results from multiple linear regression analysis, data in second row represents analysis adjusting for contemporary variables correlated with antibody response to vaccination. Data in third row represents analysis adjusting for contemporary variables correlated with antibody response to vaccination but *excluding* pre-vaccination antibody titres. Low birth weight–binary variable of subjects with a birthweight of <2.5 kg *vs*. subjects with a birth weight of ≥2.5 kg. Early-catch up growth is defined as the change in sex-specific standard deviation scores between birth and 3 months of age. Season of birth: hungry (July–December) *vs*. harvest (January–June).
